# Reduced Adherence to Antiretroviral Therapy in Pregnant Women With HIV With Intimate Partner Violence in the United States

**DOI:** 10.1093/ofid/ofaf787

**Published:** 2026-01-23

**Authors:** Aasith Villavicencio, John B Jemmott, Fatemeh Ghadimi, Hervette Nkwihoreze, Sara Seyedroudbari, William R Short, Aadia Rana, Anandi N Sheth, Rachel K Scott, Gweneth B Lazenby, Rodney L Wright, Florence M Momplaisir

**Affiliations:** Division of Infectious Diseases, Department of Medicine, Perelman School of Medicine, University of Pennsylvania, Philadelphia, Pennsylvania, USA; Division of Infectious Diseases, Department of Medicine, University of Miami Miller School of Medicine, Miami, Florida, USA; Department of Psychiatry, Perelman School of Medicine, and Annenberg School for Communication, University of Pennsylvania, Philadelphia, Pennsylvania, USA; Division of Infectious Diseases, Department of Medicine, Perelman School of Medicine, University of Pennsylvania, Philadelphia, Pennsylvania, USA; Division of Infectious Diseases, Department of Medicine, Perelman School of Medicine, University of Pennsylvania, Philadelphia, Pennsylvania, USA; College of Medicine, Drexel University, Philadelphia, Pennsylvania, USA; Division of Infectious Diseases, Department of Medicine, Perelman School of Medicine, University of Pennsylvania, Philadelphia, Pennsylvania, USA; Division of Infectious Diseases, Department of Medicine, University of Alabama-Birmingham Heersink School of Medicine, Birmingham, Alabama, USA; Department of Medicine, Division of Infectious Diseases, Emory University School of Medicine, Atlanta, Georgia, USA; MedStar Health Research Institute & MedStar Washington Hospital Center, Georgetown University School of Medicine, Washington, District of Columbia, USA; Department of Obstetrics and Gynecology, Medical University of South Carolina, Charleston, South Carolina, USA; Department of Obstetrics and Gynecology and Women's Health, Einstein College of Medicine and Montefiore Medical Center, New York, New York, USA; Division of Infectious Diseases, Department of Medicine, Perelman School of Medicine, University of Pennsylvania, Philadelphia, Pennsylvania, USA; Leonard Davis Institute of Health Economics, University of Pennsylvania, Philadelphia, Pennsylvania, USA

**Keywords:** HIV, pregnancy, intimate partner violence, antiretroviral therapy, domestic violence

## Abstract

**Background:**

Despite increased access to antiretroviral therapy (ART) for women with HIV (WWH), poor postpartum HIV care retention persists. This analysis evaluates Intimate Partner Violence (IPV) and ART adherence in pregnant WWH.

**Methods:**

We analyzed secondary data from a US behavioral intervention trial to improve postpartum retention in WWH. Data were collected from the baseline survey including the Edinburgh Postnatal Depression Scale (EPDS), adverse childhood experiences (ACE), and HIV-related stigma scores, and the WHO Violence Against Women questionnaire to assess IPV. A multivariable logistic regression examined associations between IPV timing (before, during pregnancy, any) and type (physical, psychological, sexual) and ART adherence (≥80% ART doses in the prior month).

**Results:**

A total of 137 pregnant WWH enrolled between March 2020 and March 2024 were included: mean age was 30.5 (SD 5.6); 83% were Black, 14% Hispanic; mean number of pregnancies was 3.3 (SD 2.1). Depression, stigma, and ACEs were prevalent: EPDS scores of ≥10 were seen in 45% of women, ≥4 ACEs in 23%, and 51% reported HIV-related shame. Forty women (29%) reported IPV exposure. Higher EPDS, ACE, and stigma scores were seen in women exposed to IPV (*P* < .02). Physical IPV during pregnancy had the strongest association with decreased ART adherence in pregnancy (adjusted odds ratio = 0.10, *P* = .02). Psychological IPV and any IPV type during or before pregnancy were also associated with lower odds of adherence.

**Conclusions:**

We found high IPV rates and a significant negative association with ART adherence among pregnant WWH highlighting the importance of addressing IPV in HIV care.

## Teaser

Intimate partner violence (IPV) was significantly associated with reduced antiretroviral therapy adherence among pregnant women with HIV in the US. Findings highlight the importance of addressing IPV in HIV care.

Intimatepartner violence (IPV), including physical, sexual, and psychological, has long been identified as a major public health problem affecting millions of women globally [1]. It leads to negative short- and long-term health outcomes [2–4], with adverse obstetric outcomes affecting mothers and children with IPV around the time of pregnancy [5–7]. In the US, approximately 47% of women reported any contact sexual violence, physical violence, or stalking by an intimate partner at some point in their lifetime, according to the National Intimate Partner and Sexual Violence Survey (NISVS) for 2016–2017 [8]. These rates vary across different races, ethnicities, ages, and income with some groups experiencing rates as high as 64%. Among women with HIV (WWH), the prevalence of IPV can be higher, with estimates ranging from 36% to 74% [9].

Women exposed to IPV are at increased risk of HIV acquisition [10–12]. Furthermore, IPV has been associated with HIV-related adverse outcomes albeit with mixed results, with studies suggesting women who experience IPV are more likely to report lower use of antiretroviral therapy (ART) and poorer ART adherence and are less likely to be virally suppressed [12–16]. Antiretroviral therapy adherence during pregnancy is well known to be suboptimal, with pooled estimates of only 74% of pregnant WWH reporting > 80% ART adherence globally, including women in the US [17], indicating the importance of studying possible contributing factors such as IPV.

Research regarding IPV in pregnant WWH remains scarce in the US, even in high IPV and HIV burden states [18]. We conducted a secondary analysis of data collected from pregnant WWH participating in the WISH (Women Involved in Supporting Health) trial [19] to evaluate the association between exposure to IPV and self-reported ART adherence during pregnancy in WWH in the US. We also described sociodemographic and psychosocial characteristics such as depression, stigma, and adverse childhood experiences (ACEs) during childhood or adolescence.

## MATERIALS AND METHODS

### Study Design and Participants

This study involved secondary analysis of data from WISH, a multisite US randomized controlled trial of a peer-led behavioral intervention to improve HIV care retention postpartum, long-term retention, and viral suppression. Women were recruited at the following locations across Center for AIDS research sites: Atlanta (Emory University), Charleston (Medical University of South Carolina), Washington, DC (MedStar Health Research Institute), New York City (Montefiore Medical Center/Albert Einstein College of Medicine), Birmingham (the University of Alabama at Birmingham), and Philadelphia (the University of Pennsylvania). The participating HIV clinics at these sites receive support from the Ryan White HIV/AIDS Program (RWHAP, administered by the Health Resources and Services Administration in the US Department of Health and Human Services), which provides HIV primary care, support services, and medication to low-income, uninsured, or otherwise underserved populations living with HIV in safety net settings. Eligible participants were cisgender women with HIV aged 16 years or older, in the second or third trimester of pregnancy, with access to a cell phone, and able to read and speak English and provide informed consent. Women with plans to relocate outside the US within the year following delivery were excluded. Potential participants were informed of the study by treating physicians or study team members. A site coordinator followed up with the women interested in participating in the study to further screen for eligibility and enroll them. For the present article, we used only baseline survey data, which was collected once after enrollment and before the intervention (during pregnancy). The analysis included participants enrolled as of March 2024.

### Measures

The baseline survey consisted of self-reported data collected using audio computer-assisted self-interviewing (ACASI). Variables included sociodemographic, clinical, and psychosocial characteristics using already established instruments such as the Edinburgh Postnatal Depression Scale (EPDS), ACEs, an HIV-related stigma score, and the World Health Organization (WHO) Violence Against Women Questionnaire (survey questions provided in Supplement). The EPDS, a depression screening tool widely used in pregnant and postpartum women, contains 10 questions, each scored on a 4-point Likert scale (0–3), yielding a total score ranging from 0 to 30, with higher scores indicating more severe symptoms [20, 21]. We reported the mean EPDS score and further dichotomized this variable into 0–9 (negative screening) and ≥10 (positive screening) for the descriptive analysis, as the latter cutoff has been shown to have high sensitivity and specificity and identify most patients with fewer false negatives [22]. Adverse childhood experiences were assessed through an 11-item questionnaire, which the United States Centers for Disease Control and Prevention used as part of their Behavioral Risk Factor Surveillance System survey [23, 24], adapted from the ACE study on adversity experienced before age 18 [25]. Adverse childhood experiences include exposure to direct harm (emotional, physical, sexual abuse) as well as household adversity like parental separation or divorce, mental illness, substance use, domestic violence, and incarcerated family members [23, 24]. Adverse childhood experience scores were calculated by totaling the 11 survey items, with 1 point assigned for each affirmative response (range 0–11). A greater number of ACEs has been associated with increased odds of psychiatric disorders during adulthood, including depression, PTSD, and substance use [26–28], as well as physical illnesses like cancer and cardiovascular disease [27]. We dichotomized the ACE score variable into 0–3 and ≥4, given that reported exposure to 4 or more ACEs has been linked to an increased likelihood of adverse health outcomes in multiple studies [29–31]. An HIV-related stigma score was derived from 9 questions from Stangl's “Health Stigma and Discrimination Framework,” [32] which contains questions from The People Living with HIV Stigma Index [33]. One point was assigned for each “yes” response related to experiencing internalized or social stigma (range 0–9).

Exposure to IPV by their primary partner was assessed using the WHO Violence Against Women Questionnaire [34, 35], adapted to include 12 items, and the timing of IPV. Physical violence was defined as experiencing any of the following: being slapped or having something thrown at her that could hurt her; being pushed or shoved; being hit, kicked, or dragged; being beaten; or being choked or burned. Psychological IPV included being insulted or made to feel bad about herself, belittled or humiliated in front of other people, scared or intimidated on purpose, being threatened to hurt her or someone she cared about. Sexual violence included being threatened to have sex, being physically forced to have sex, or being forced to do something sexual that she found degrading or humiliating. Responses for each item were originally collected on a frequency scale and dichotomized into any exposure to physical, psychological, or sexual IPV versus no exposure (never). We further categorized exposure to IPV according to timing: before current pregnancy, during current pregnancy, or any.

As in prior studies, ART adherence was defined as ≥ 80% of doses taken as prescribed over the past 30 days, as assessed by self-report. This threshold has previously been used in other studies and has been deemed sufficient to achieve viral suppression with modern ART [17, 36–39].

### Data Analysis

Sociodemographic, clinical, and psychosocial characteristics were analyzed using descriptive statistics (eg, frequencies, percentages, mean, and median). A multivariable logistic regression (MLR) was used to examine associations between IPV exposure at different times (before pregnancy, during pregnancy, or any exposure) and types (psychological, physical, or sexual) and ART adherence in pregnancy (≥80% of ART doses in the prior 30 days), estimating adjusted odds ratios (aOR). Covariates found to have an association with both IPV and ART adherence in previous studies were included in the MLR: age, Hispanic ethnicity, education, employment status, EPDS score, HIV-related stigma score, and substance use (tobacco, alcohol, drug use) during and before pregnancy. The EPDS and HIV-related stigma scores were accounted for quantitatively, and the rest of the variables were reduced to 2 categories for the MLR. Adverse childhood experience scores were excluded from the final MLR model due to significant multicollinearity with the IPV variables.

All analyses were conducted using R statistical software, version 4.3.0 (R Foundation for Statistical Computing), and statistical tests were 2-tailed with *P* < .05. Data were analyzed from April through June 2024.

### Ethical Considerations

Ethical approval for this study was obtained from the Institutional Review Board of the University of Pennsylvania and Penn Medicine (number 833893), who reviewed and approved the protocol. The study was conducted in line with the principles of the World Medical Association's Declaration of Helsinki. The participants provided written informed consent to participate in this study.

### Role of the Funding Source

The WISH study was funded by the National Institute on Minority Health and Health Disparities (NIMHD), grant number R01MD013558-01; clinical trial registration NCT04168008. The funder had no role in the study design, data collection, analysis, interpretation, or report writing.

## RESULTS

### Sociodemographic Characteristics

A total of 137 pregnant WWHs enrolled between March 2020 and March 2024 were enrolled. The mean age was 30.5 (SD 5.6). Most women were Black (114, 83.2%); 11 (8.8%), White; 12 (8%), other race. Hispanic ethnicity was reported by 19 (13.9%) of women, and 20 (14.6%) were born outside the US. Most women reported having a relationship with a male partner for 6 months or longer (108, 78.8%) and 86 (62.8%) for 1 year or longer. The mean relationship duration for the 86 women with partnerships longer than a year was 6.4 (SD 6.3) years. Amongst women with relationships longer than 6 months, nearly half were living with their partner (46/108, 42.6%), most had disclosed their HIV status to their partner (88/108, 81.5%), and 18 women had partners with HIV. Women who did not disclose their HIV status reported fear of abandonment (14/20) and fear of physical violence (2/20). Most women (111, 81%) reported ART adherence to ≥ 80% of doses in the past 30 days. Further sociodemographic, medical history, social history, and partner-related characteristics are presented in Table 1.

**Table 1.** Participant Characteristics and Prevalence of IPV During and Before Pregnancy Among Pregnant WWH (N = 137)

Characteristics	Total (n = 137)	Any IPV (n = 40)	No IPV (n = 97)	*P V*alue	IPV During Pregnancy (n = 33)	No IPV During Pregnancy (n = 104)	*P V*alue	IPV Before Pregnancy (n = 26)	No IPV Before Pregnancy (n = 111)	*P V*alue
*Sociodemographic*										
Age, years—mean (SD)	30.5 (5.6)	29.9 (5.3)	30.7 (5.8)	.45	29.3 (4.8)	30.9 (5.8)	.14	30.2 (5.8)	30.6 (5.6)	.75
Hispanic or Latino Ethnicity—n (%)	19 (13.9)	3 (7.5)	16 (16.5)	.27	3 (7.5)	16 (16.5)	.53	1 (3.9)	18 (16.2)	.18
Race—n (%)										
Black	114 (83.2)	32 (80)	82 (84.5)	.80	25 (75.8)	89 (85.6)	.42	22 (84.6)	92 (82.9)	.97
White	11 (8.8)	4 (10)	7 (7.2)		4 (12.1)	7 (6.7)		2 (7.7)	9 (8.1)	
Other	12 (8)	4 (10)	8 (8.3)		4 (12.1)	8 (7.7)		2 (7.7)	10 (9)	
Foreign-born—n (%)	20 (14.6)	5 (12.5)	15 (15.5)	.86	4 (12.1)	16 (15.4)	.86	3 (11.5)	17 (15.3)	.86
Marital status—n (%)										
Married	20 (14.6)	4 (10)	16 (16.5)	.50	4 (12.1)	16 (15.4)	.89	2 (7.7)	18 (16.2)	.48
Never married	104 (75.9)	33 (82.5)	71 (73.2)		26 (78.8)	78 (75)		22 (84.6)	82 (73.9)	
Widowed/Divorced/Separated	13 (9.5)	3 (7.5)	10 (10.3)		3 (9.1)	10 (9.6)		2 (7.7)	11 (9.9)	
Educational level—n (%)										
Primary/high school or GED	85 (62)	24 (60)	61 (62.9)	.90	20 (60.6)	65 (62.5)	1	12 (46.2)	73 (65.8)	.10
Tertiary	52 (38)	16 (40)	36 (37.1)		13 (39.4)	39 (37.5)		14 (53.8)	38 (34.2)	
Employed—n (%)	57 (41.6)	18 (45)	39 (40.2)	.74	15 (45.5)	42 (40.4)	.75	13 (50)	44 (39.6)	.46
*Clinical characteristics*										
Pregnancies including the current one—mean (SD)	3.31 (2.1)	3.28 (1.9)	3.33 (2.1)	.88	3.21 (1.8)	3.35 (2.1)	.72	3.23 (1.8)	3.33 (2.1)	.80
Unintended pregnancy—n (%)	100 (72.9)	33 (82.5)	67 (69.1)	.16	28 (84.8)	72 (69.2)	.12	22 (84.6)	78 (70.3)	.22
EPDS score—mean (SD)^a^	8.5 (5.5)	11.8 (4.8)	7.1 (5.1)	.00	11.8 (4.6)	7.5 (5.3)	.00	12 (5.1)	7.7 (5.2)	.00
EPDS score ≥10—n (%)^a^	62 (45.3)	30 (75)	32 (32.9)	.00	25 (75.8)	37 (35.6)	.00	20 (76.9)	42 (37.8)	.00
Years since HIV diagnosis—mean (SD)^a^	9.26 (8.7)	10.6 (9.9)	8.7 (8.2)	.29	9.7 (9.4)	9.1 (8.5)	.79	9.5 (9.8)	9.2 (8.5)	.87
HIV diagnosis in current pregnancy—n (%)	33 (24.1)	8 (20)	25 (25.8)	.62	6 (18.2)	27 (25.9)	.49	7 (26.9)	26 (23.4)	.90
Currently taking ART—n (%)	132 (96.4)	38 (95)	94 (96.9)	.97	32 (96.9)	100 (96.2)	1	25 (96.2)	107 (96.4)	1
ART dose percentage, past 30 d—mean (SD)	88.6 (22.2)	82.4 (26.4)	91.2 (19.8)	.06	81.7 (25.2)	90.8 (20.8)	.07	77.9 (30.6)	91.1 (19.0)	.04
Adherence ≥80% of ART doses, past 30 d—n (%)	111 (81)	28 (70)	83 (85.6)	.06	22 (66.7)	89 (85.6)	.03	17 (65.4)	94 (70.7)	.05
*Partner/psychosocial characteristics^b^*										
ACE score—mean (SD)	1.9 (1.9)	2.7 (2.0)	1.5 (1.8)	.00	2.6 (2.0)	1.6 (1.8)	.02	3.0 (1.9)	1.6 (1.8)	.00
ACE score ≥ 4—n (%)	32 (23.3)	14 (35)	18 (18.6)	.06	12 (36.4)	20 (19.2)	.07	11 (42.3)	21 (18.9)	.02
Relationship for 6 m or longer—n (%)	108 (78.8)	24 (60)	84 (86.6)	.00	21 (63.6)	87 (83.7)	.03	16 (61.5)	92 (82.9)	.03
Living with partner—n (%)	46 (33.6)	10 (25)	36 (37.1)	.24	10 (30.3)	36 (34.6)	.81	6 (23.1)	40 (36)	.30
Partner's HIV positive status—n (%)	18 (16.6)	3 (7.5)	15 (15.5)	.33	3 (9.1)	15 (14.4)	.62	2 (7.7)	16 (14.4)	.55
Reported HIV-related shame, past year—n (%)	69 (50.7)	26 (65)	43 (44.3)	.05	21 (63.7)	48 (46.2)	.13	19 (73.1)	50 (45)	.02
HIV-related stigma score—mean (SD)	2.7 (2.4)	3.6 (2.3)	2.3 (2.3)	.01	3.22 (2.2)	2.5 (2.4)	.12	3.9 (2.1)	2.4 (2.3)	.00
Tobacco use during pregnancy—n (%)	29 (21)	14 (35)	15 (15.5)	.02	12 (36.4)	17 (16.3)	.03	8 (30.8)	21 (18.9)	.29
Tobacco use before pregnancy—n (%)	51 (37.2)	18 (45)	33 (34)	.31	13 (39.4)	38 (36.5)	.93	12 (46.2)	39 (35.1)	.41
Alcohol use during pregnancy^c^—n (%)	9 (6.5)	3 (7.5)	6 (6.2)	1	2 (6)	7 (6.7)	1	2 (7.7)	7 (6.3)	1
Alcohol use before pregnancy^c^—n (%)	52 (37.9)	19 (47.5)	33 (34)	.19	15 (45.5)	37 (35.6)	.42	13 (50)	93 (83.8)	.24
Illicit drug use during pregnancy^d^—n (%)	39 (28.5)	18 (45)	21 (21.6)	.01	13 (39.4)	26 (25)	.17	13 (50)	26 (23.4)	.01
Illicit drug use before pregnancy^d^—n (%)	56 (40.9)	21 (52.5)	35 (36)	.11	16 (48.5)	40 (38.5)	.41	14 (53.8)	42 (37.8)	.20

Abbreviations: ACE, adverse childhood experience; EPDS, Edinburgh Postnatal Depression Scale; IPV, intimate partner violence.

^a^Data missing for 1 participant.

^b^All the participants who reported being in a relationship reported their partners were male.

^c^Alcohol use was defined as 4 or more drinks in a day.

^d^Illicit drug use included cannabis, cocaine, opioids, or other illegal drug use.

### Burden of Depression, Stigma, and Adverse Childhood Experiences

An EPDS score of ≥ 10, indicating positive screening for depression, was seen in 45% of women, and a high ACE score (≥4) in 23% (Table 1). More than half the women (51%) reported HIV-related shame, with a mean score of 2.7 (SD 2.4). In a bivariate analysis, significantly higher EPDS, ACE, and stigma scores were seen in women exposed to IPV (*P* < .02) (Table 1).

### Association Between IPV and ART Adherence: Multivariable Logistic Regression

**Figure 1.**
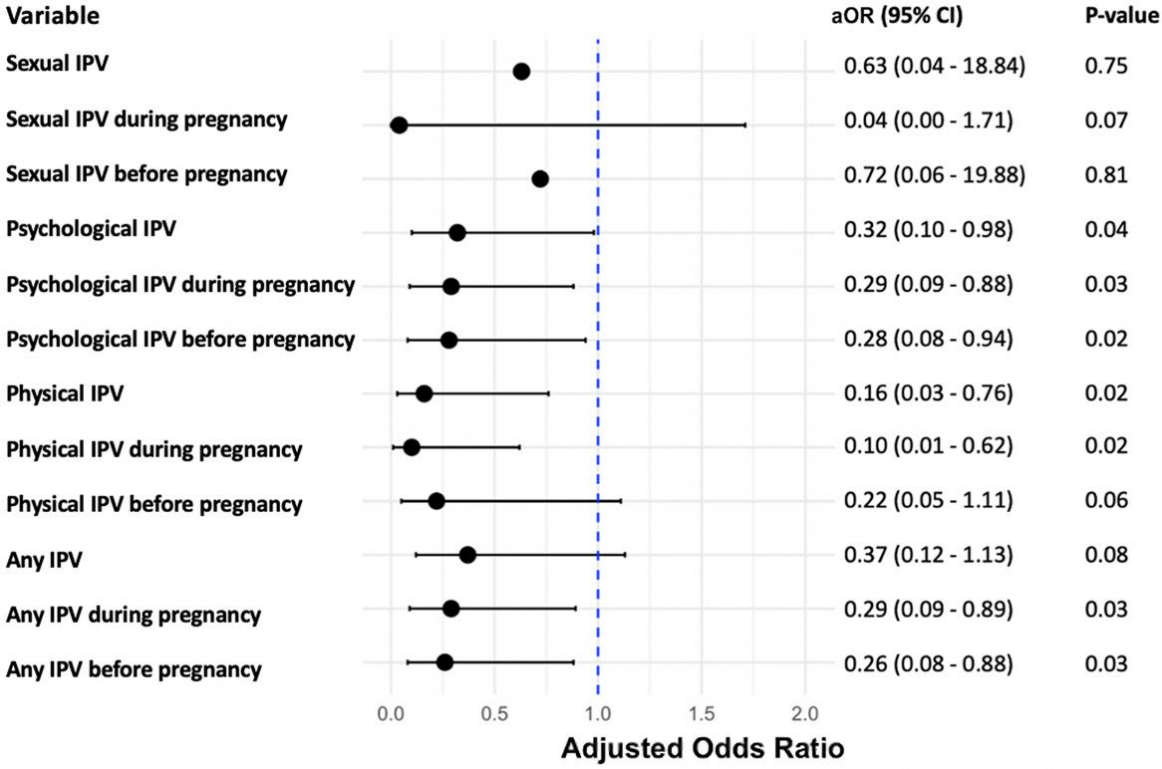
Forest Plot and Multivariable Logistic Regression of the Association Between IPV and ART Adherence during Pregnancy (N = 137). ^a^Reference category for intimate partner violence (IPV): no exposure to IPV. Sexual IPV, psychological IPV, physical IPV, and any IPV represent exposure during pregnancy or before pregnancy. ^b^Adjusted for age, Hispanic ethnicity, education, employment status, the Edinburgh Postnatal Depression Scale (EPDS) score, the HIV-related stigma score, and substance use (tobacco, alcohol, drug use) during and before pregnancy (adjustment for before pregnancy only applies to IPV before pregnancy). ^c^Sexual IPV results are presented with caution due to small sample sizes and large confidence intervals. The forest plot excluded outliers for confidence intervals beyond 0 to 2 for clarity and readability. Abbreviations: aOR, adjusted odds ratio; ART, antiretroviral therapy; CI, confidence interval; IPV, intimate partner violence.

Forty women (29%) reported IPV exposure at any point before or during pregnancy (39, psychological; 13, physical; 4, sexual). After adjusting for sociodemographic factors, EPDS, and HIV-stigma scores in the multivariable logistic regression, exposure to physical IPV during pregnancy had the strongest association with decreased ART adherence in pregnancy (aOR = 0.10, *P* = .02) (Figure 1). Exposure to psychological IPV or any IPV type during or before pregnancy was also significantly associated with lower odds of ART adherence in pregnancy (Figure 1).

## DISCUSSION

Our study showed a high rate of IPV (29%) and a significant association between exposure to IPV and decreased ART adherence in pregnancy in WWH from different sites in the United States, with the strongest association seen with physical IPV. Our study population also had a high prevalence of depression, HIV-related stigma, and ACEs, which were further quantified with validated scores.

Single studies evaluating the association between IPV and ART adherence in WWH have shown mixed results. Hatcher et al. conducted a meta-analysis of 13 studies and found that IPV was associated with 21% lower odds of current ART use and 52% lower odds of self-reported ART adherence in WWH; however, significant variability in sampling and assessment tools limited the ability to generalize these findings. Women reporting IPV were also less likely to have viral suppression. Similarly, a pooled analysis of population-based data from Sub-Saharan Africa showed inconclusive results for the association between lifetime and past-year physical or sexual IPV with self-reported ART uptake and adherence but a clear association between IPV and “viral load suppression,” defined by HIV RNA level below 1000 copies/mL [12] as opposed to optimal viral suppression defined as HIV RNA below the lower limit of detection (generally <20 copies/mL) [40].

The intersection of ART adherence and IPV during pregnancy and postpartum significantly impacts maternal and child health. Still, research in this area remains limited and comes mostly from low- and middle-income countries. Studies in South Africa have shown a link between IPV and decreased ART adherence in WWH during pregnancy [41] and in the postpartum period [42]. To our knowledge, only one study by Yee et al. [43] evaluated this association in the US; 14% of pregnant WWH in a multidisciplinary clinic experienced IPV during pregnancy. IPV was further associated with a longer time to viral suppression after starting ART, although no link was established with self-reported ART adherence [43]. A meta-analysis, including Yee's study, did not find an association between ART nonadherence and IPV in pregnant WWH but did find an association between non-adherence and infant antiretroviral prophylaxis based on 2 studies [42, 44, 45]. Furthermore, exposure to IPV has been linked to non-disclosure of HIV status among postpartum WWH [46, 47].

Our study also found significantly higher EPDS, ACE, and stigma scores in women exposed to IPV (*P* < .02). It is well known that perinatal depression and ACEs can lead to negative outcomes for mothers and infants [48, 49]. Pregnant WWH often experience high rates of depression [50], which is linked to decreased adherence to ART and lower rates of viral suppression [51]. In South Africa, more ACEs correlate with suboptimal adherence and elevated HIV RNA levels (≥1000 copies/mL) [52]. Moreover, HIV-related stigma has been linked to lower odds of viral suppression in people with HIV (PWH) [53] across genders and particularly among African American WWH [54, 55], with the association seen even without depression as a mediator [55].

The relationship between IPV, perinatal depression, and ACEs is complex. A meta-analysis shows that higher ACE scores (before 18 years of age) correlate with IPV, as a perpetrator and a victim, with a stronger association among younger individuals [56]. Depression has been studied both as a result of IPV as well as a mediator between ACEs and IPV [57]. In pregnant WWH, higher ACE scores correlate with an increased risk of depressive symptoms [58]. Furthermore, internalized stigma and major depression are closely intertwined in postpartum WWH [59], and IPV is associated with depression symptoms, both prenatal and postnatal [60–64], alongside stigma [61] in pregnant WWH.

Our findings highlight the urgent need for efforts to prevent, screen, and address IPV in pregnant WWH to improve maternal and child health outcomes, including the elimination of vertical transmission of HIV. A recent study indicated that 1 in 8 new infant HIV infections could have been prevented through IPV elimination in African countries with high IPV and HIV burden, linking IPV to ART uptake [65]. Improved diagnosis and treatment of depression could also increase viral suppression rates by nearly 5% among PWH, per modeling analyses [66]. US guidelines recommend counseling to prevent prenatal depression [67] and screening for depression and IPV among pregnant women [68–73], including pregnant WWH [74]. While current depression interventions are effective in PWH [75, 76], there is a lack of evidence-based approaches addressing mental health, IPV, and stigma during the perinatal period in WWH [77, 78]. Research on IPV, particularly in high-resource settings, is particularly lagging. US guidelines also recommend referrals for supportive care for IPV among pregnant WWH when indicated [74]. Interventions that prevent and respond to IPV should be evaluated and integrated into HIV care of pregnant and postpartum women by using existing multidisciplinary models that incorporate mental health and social services [79]. These efforts should involve healthcare workers, researchers, national and global policymakers, and communities, considering social determinants of health. It is essential to prioritize populations with a high burden of HIV and IPV, such as those in the southern US [18, 80] and racial and ethnic minorities disproportionally affected by HIV [81], especially black women who face the highest maternal death risk due to disparities [82]. Additionally, research on the role of ACE screening in prenatal care could be beneficial in guiding trauma-informed care [83] for WWH.

### Strengths and Limitations

Our study has several strengths as it contributes insights into the intersection of IPV and ART adherence among pregnant WWH from high-resource settings and an underexplored area in the US. Validated tools were used, including the EPDS, ACEs questionnaire, and the WHO's Violence Against Women questionnaire. Additionally, we informed on other psychosocial factors such as depression, stigma, and ACEs that are interconnected with IPV and HIV outcomes.

Limitations of the study include ART adherence assessment by self-report, which may be subject to social desirability and recall bias. While additional viral load data would have been useful to support the self-reported data, our study is reflective of the patient-level clinical context in which providers often counsel patients. Moreover, while adherence levels needed for viral suppression can be regimen-dependent [37] and higher levels of adherence (100%) are associated with improved outcomes beyond viral suppression [84], using a threshold of ≥80% more conservatively allows imperfect recall and reporting. Additionally, the sample is drawn from a limited number of sites that may not represent all pregnant WWH populations and did not include speakers of languages other than English, limiting the generalizability of the results. Given the cross-sectional nature of the survey, causal relationships or long-term trends could not be established. Lastly, although cofounders such as substance use were included, ACEs were excluded from the final regression model due to multicollinearity, thus potentially affecting the findings.

## CONCLUSIONS

This study highlights the critical intersection of IPV and HIV, with a strong association seen between IPV and ART adherence in pregnant WWH. Our findings underscore the need to adhere to IPV screening recommendations in prenatal care and the importance of addressing IPV as part of HIV care, especially when coupled with depression, stigma, or high ACE scores in pregnant women with HIV.

## Supplementary Material

ofaf787_Supplementary_Data

## Supplementary Data

Supplementary materials are available at *Open Forum Infectious Diseases* online. Consisting of data provided by the authors to benefit the reader, the posted materials are not copyedited and are the sole responsibility of the authors, so questions or comments should be addressed to the corresponding author.

## Abbreviations

aOR: Adjusted odds ratio
ACASI: Audio computer-assisted self-interviewing
ACE: Adverse Childhood Experience
EPDS: Edinburgh Postnatal Depression Scale
IPV: Intimate partner violence
PWH: People with HIV
WHO: World Health Organization
WWH: Women with HIV

## References

[1] Sardinha L, Maheu-Giroux M, Stöckl H, Meyer SR, García-Moreno C. Global, regional, and national prevalence estimates of physical or sexual, or both, intimate partner violence against women in 2018. Lancet 2022; 399:803–13. 35182472 10.1016/S0140-6736(21)02664-7 PMC8885817

[2] Mellar BM, Hashemi L, Selak V, Gulliver PJ, McIntosh TKD, Fanslow JL. Association between Women's exposure to intimate partner violence and self-reported health outcomes in New Zealand. JAMA Netw Open 2023; 6:e231311. 36867408 10.1001/jamanetworkopen.2023.1311 PMC9984970

[3] Stubbs A, Szoeke C. The effect of intimate partner violence on the physical health and health-related behaviors of women: a systematic review of the literature. Trauma Violence Abuse 2022; 23:1157–72. 33541243 10.1177/1524838020985541

[4] White SJ, Sin J, Sweeney A, et al Global prevalence and mental health outcomes of intimate partner violence among women: a systematic review and meta-analysis. Trauma Violence Abuse 2024; 25:494–511. 36825800 10.1177/15248380231155529 PMC10666489

[5] Alhusen JL, Ray E, Sharps P, Bullock L. Intimate partner violence during pregnancy: maternal and neonatal outcomes. J Womens Health (Larchmt) 2015; 24:100–6. 25265285 10.1089/jwh.2014.4872 PMC4361157

[6] Hahn CK, Gilmore AK, Aguayo RO, Rheingold AA. Perinatal intimate partner violence. Obstet Gynecol Clin North Am 2018; 45:535–47. 30092927 10.1016/j.ogc.2018.04.008 PMC6089231

[7] Mead JS, Pollack CC, Paris AE, Emeny RT, Puleo RA, St Ivany AR. Obstetric outcomes among women with a history of intimate partner violence in the United States. Obstet Gynecol 2023; 142:80–9. 37290101 10.1097/AOG.0000000000005216 PMC10330567

[8] Smith SG, Zhang X, Basile KC, et al The National Intimate Partner and Sexual Violence Survey: 2015 data brief—updated release. **2018**. Accessed October 23, 2024. Available at: https://stacks.cdc.gov/view/cdc/60893.

[9] Machtinger EL, Wilson TC, Haberer JE, Weiss DS. Psychological trauma and PTSD in HIV-positive women: a meta-analysis. AIDS Behav 2012; 16:2091–100. 22249954 10.1007/s10461-011-0127-4

[10] Jewkes RK, Dunkle K, Nduna M, Shai N. Intimate partner violence, relationship power inequity, and incidence of HIV infection in young women in South Africa: a cohort study. Lancet 2010; 376:41–8. 20557928 10.1016/S0140-6736(10)60548-X

[11] Kouyoumdjian FG, Calzavara LM, Bondy SJ, et al Intimate partner violence is associated with incident HIV infection in women in Uganda. AIDS 2013; 27:1331–8. 23925380 10.1097/QAD.0b013e32835fd851

[12] Kuchukhidze S, Panagiotoglou D, Boily M-C, et al The effects of intimate partner violence on women's risk of HIV acquisition and engagement in the HIV treatment and care cascade: a pooled analysis of nationally representative surveys in sub-Saharan Africa. Lancet HIV 2023; 10:e107–17. 36463914 10.1016/S2352-3018(22)00305-8

[13] Hatcher AM, Smout EM, Turan JM, Christofides N, Stöckl H. Intimate partner violence and engagement in HIV care and treatment among women: a systematic review and meta-analysis. AIDS 2015; 29:2183–94. 26353027 10.1097/QAD.0000000000000842

[14] Rose RC, House AS, Stepleman LM. Intimate partner violence and its effects on the health of African American HIV-positive women. Psychol Trauma 2010; 2:311–7.

[15] Siemieniuk RAC, Krentz HB, Miller P, Woodman K, Ko K, Gill MJ. The clinical implications of high rates of intimate partner violence against HIV-positive women. J Acquir Immune Defic Syndr 2013; 64:32–8. 23714742 10.1097/QAI.0b013e31829bb007

[16] Trimble DD, Nava A, McFarlane J. Intimate partner violence and antiretroviral adherence among women receiving care in an urban Southeastern Texas HIV clinic. J Assoc Nurses AIDS Care 2013; 24:331–40. 23790276 10.1016/j.jana.2013.02.006

[17] Nachega JB, Uthman OA, Anderson J, et al Adherence to antiretroviral therapy during and after pregnancy in low-income, middle-income, and high-income countries: a systematic review and meta-analysis. AIDS 2012; 26:2039–52. 22951634 10.1097/QAD.0b013e328359590f PMC5061936

[18] Johnson KA, Binion S, Waller B, et al Left behind in the U.S.’ deep south: addressing critical gaps in HIV and intimate partner violence prevention efforts targeting black women. Front Reprod Health 2022; 4:1008788. 36505393 10.3389/frph.2022.1008788 PMC9733498

[19] NIH RePORT . A Randomized Controlled Trial of Women Involved in Supporting Health (WISH), a peer-led intervention to improve postpartum retention in HIV care. Bethesda, MD: National Institute on Minority Health and Health Disparities, 2024. Accessed May 8, 2025. Available at: https://reporter.nih.gov/project-details/10372917.

[20] Cox JL, Holden JM, Sagovsky R. Detection of postnatal depression. Development of the 10-item Edinburgh Postnatal Depression Scale. Br J Psychiatry 1987; 150:782–6. 3651732 10.1192/bjp.150.6.782

[21] O’Connor E, Rossom RC, Henninger M, Groom HC, Burda BU. Primary care screening for and treatment of depression in pregnant and postpartum women: evidence report and systematic review for the US Preventive Services Task Force. JAMA 2016; 315:388–406. 26813212 10.1001/jama.2015.18948

[22] Levis B, Negeri Z, Sun Y, Benedetti A, Thombs BD. Accuracy of the Edinburgh Postnatal Depression Scale (EPDS) for screening to detect major depression among pregnant and postpartum women: systematic review and meta-analysis of individual participant data. BMJ 2020; 371:m4022. 33177069 10.1136/bmj.m4022 PMC7656313

[23] Aslam MV, Swedo E, Niolon PH, Peterson C, Bacon S, Florence C. Adverse childhood experiences among U.S. Adults: national and state estimates by adversity type, 2019–2020. Am J Prev Med 2024; 67:55–66. 38369270 10.1016/j.amepre.2024.02.010 PMC11193602

[24] Centers for Disease Control and Prevention. Behavioral Risk Factor Surveillance System (BRFSS) Adverse Childhood Experiences (ACE) module. Atlanta, GA: U.S. Department of Health and Human Services, Centers for Disease Control and Prevention , 2021. Accessed August 5, 2024.

[25] Felitti VJ, Anda RF, Nordenberg D, et al Relationship of childhood abuse and household dysfunction to many of the leading causes of death in adults. The Adverse Childhood Experiences (ACE) study. Am J Prev Med 1998; 14:245–58. 9635069 10.1016/s0749-3797(98)00017-8

[26] Daníelsdóttir HB, Aspelund T, Shen Q, et al Adverse childhood experiences and adult mental health outcomes. JAMA Psychiatry 2024; 81:586–94. 38446452 10.1001/jamapsychiatry.2024.0039 PMC10918580

[27] Hughes K, Bellis MA, Hardcastle KA, et al The effect of multiple adverse childhood experiences on health: a systematic review and meta-analysis. Lancet Public Health 2017; 2:e356–66. 29253477 10.1016/S2468-2667(17)30118-4

[28] Liu M, Luong L, Lachaud J, Edalati H, Reeves A, Hwang SW. Adverse childhood experiences and related outcomes among adults experiencing homelessness: a systematic review and meta-analysis. Lancet Public Health 2021; 6:e836–47. 34599894 10.1016/S2468-2667(21)00189-4

[29] Bellis MA, Lowey H, Leckenby N, Hughes K, Harrison D. Adverse childhood experiences: retrospective study to determine their impact on adult health behaviours and health outcomes in a UK population. J Public Health 2013; 36:81–91. 10.1093/pubmed/fdt038 23587573

[30] Merrick MT, Ford DC, Ports KA, et al Vital signs: estimated proportion of adult health problems attributable to adverse childhood experiences and implications for prevention—25 states, 2015–2017. MMWR Morb Mortal Wkly Rep 2019; 68:999–1005. 31697656 10.15585/mmwr.mm6844e1 PMC6837472

[31] Yu J, Patel RA, Haynie DL, et al Adverse childhood experiences and premature mortality through mid-adulthood: a five-decade prospective study. Lancet Reg Health Am 2022; 15:100349. 36467261 10.1016/j.lana.2022.100349 PMC9718480

[32] Stangl AL, Brady L, Fritz K. Measuring HIV stigma and discrimination. Washington, DC: International Center for Research on Women, 2012:1–2.

[33] Friedland BA, Gottert A, Hows J, et al The people living with HIV stigma Index 2.0: generating critical evidence for change worldwide. AIDS 2020; 34 Suppl 1:S5–s18. 32881790 10.1097/QAD.0000000000002602 PMC11758485

[34] Garcia-Moreno C, Jansen HAFM, Ellsberg M, Heise L, Watts CH. Prevalence of intimate partner violence: findings from the WHO multi-country study on women's health and domestic violence. Lancet 2006; 368:1260–9. 17027732 10.1016/S0140-6736(06)69523-8

[35] World Health Organization . WHO multi-country study on women's Health and domestic violence against women: initial results on prevalence, health outcomes and women's Responses. Geneva, Switzerland: World Health Organization, 2005.

[36] Bezabhe WM, Chalmers L, Bereznicki LR, Peterson GM. Adherence to antiretroviral therapy and virologic failure: a meta-analysis. Medicine (Baltimore) 2016; 95:e3361. 27082595 10.1097/MD.0000000000003361 PMC4839839

[37] Byrd KK, Hou JG, Hazen R, et al Antiretroviral adherence level necessary for HIV viral suppression using real-world data. J Acquir Immune Defic Syndr 2019; 82:245–51. 31343455 10.1097/QAI.0000000000002142 PMC6854523

[38] O'Halloran Leach E, Lu H, Caballero J, Thomas JE, Spencer EC, Cook RL. Defining the optimal cut-point of self-reported ART adherence to achieve viral suppression in the era of contemporary HIV therapy: a cross-sectional study. AIDS Res Ther 2021; 18:36. 34174904 10.1186/s12981-021-00358-8 PMC8234726

[39] Viswanathan S, Detels R, Mehta SH, Macatangay BJ, Kirk GD, Jacobson LP. Level of adherence and HIV RNA suppression in the current era of highly active antiretroviral therapy (HAART). AIDS Behav 2015; 19:601–11. 25342151 10.1007/s10461-014-0927-4 PMC4393774

[40] Centers for Disease Control and Prevention; HIV Medicine Association of the Infectious Diseases Society of America; Pediatric Infectious Diseases Society; HHS Panel on Treatment of HIV During Pregnancy and Prevention of Perinatal Transmission A Working Group of the Office of AIDS Research Advisory Council (OARAC). Panel on antiretroviral guidelines for adults and adolescents. Guidelines for the use of antiretroviral agents in adults and adolescents with HIV 2024. Washington DC: U.S. Department of Health and Human Services, **2024**. Accessed November 1, 2024. Available at: https://clinicalinfo.hiv.gov/en/guidelines/adultand-adolescent-arv.

[41] Ramlagan S, Peltzer K, Ruiter RAC, Barylski NA, Weiss SM, Sifunda S. Prevalence and factors associated with fixed-dose combination antiretroviral drugs adherence among HIV-positive pregnant women on option B treatment in Mpumalanga Province, South Africa. Int J Environ Res Public Health 2018; 15:161. 29361675 10.3390/ijerph15010161 PMC5800260

[42] Hampanda KM . Intimate partner violence and HIV-positive women's non-adherence to antiretroviral medication for the purpose of prevention of mother-to-child transmission in Lusaka, Zambia. Soc Sci Med 2016; 153:123–30. 26896876 10.1016/j.socscimed.2016.02.011 PMC4788551

[43] Yee LM, Crisham Janik M, Dorman RM, Chong PS, Garcia PM, Miller ES. Relationship between intimate partner violence and antiretroviral adherence and viral suppression in pregnancy. Sex Reprod Healthc 2018; 17:7–11. 30193723 10.1016/j.srhc.2018.05.001

[44] Cook RR, Peltzer K, Weiss SM, Rodriguez VJ, Jones DL. A Bayesian analysis of prenatal maternal factors predicting nonadherence to infant HIV medication in South Africa. AIDS Behav 2018; 22:2947–55. 29302843 10.1007/s10461-017-2010-4 PMC6034978

[45] Lin D, Zhang C, Shi H. Adverse impact of intimate partner violence against HIV-positive women during pregnancy and post-partum: results from a meta-analysis of observational studies. Trauma Violence Abuse 2023; 24:1624–39. 35258353 10.1177/15248380211073845

[46] Kiarie JN, Farquhar C, Richardson BA, et al Domestic violence and prevention of mother-to-child transmission of HIV-1. AIDS 2006; 20:1763–9. 16931941 10.1097/01.aids.0000242823.51754.0c PMC3384736

[47] Kinuthia J, Singa B, McGrath CJ, et al Prevalence and correlates of non-disclosure of maternal HIV status to male partners: a national survey in Kenya. BMC Public Health 2018; 18:671. 29848345 10.1186/s12889-018-5567-6 PMC5975408

[48] Mamun A, Biswas T, Scott J, et al Adverse childhood experiences, the risk of pregnancy complications and adverse pregnancy outcomes: a systematic review and meta-analysis. BMJ Open 2023; 13:e063826. 10.1136/bmjopen-2022-063826 PMC10401231 37536966

[49] O’Connor ESC, Henninger M, Gaynes BN, Coppola E, Soulsby MW. Interventions to prevent perinatal depression: a systematic evidence review For the US preventive services task force: evidence synthesis no. 172. Rockville, MD: Agency for Healthcare Research and Quality; **2019**. AHRQ publication no. 18-05243-EF-1.

[50] Kapetanovic S, Dass-Brailsford P, Nora D, Talisman N. Mental health of HIV-seropositive women during pregnancy and postpartum period: a comprehensive literature review. AIDS Behav 2014; 18:1152–73. 24584458 10.1007/s10461-014-0728-9 PMC4120872

[51] Momplaisir F, Hussein M, Kacanek D, et al Perinatal depressive symptoms, Human Immunodeficiency Virus (HIV) suppression, and the underlying role of antiretroviral therapy adherence: a longitudinal mediation analysis in the IMPAACT P1025 cohort. Clin Infect Dis 2021; 73:1379–87. 33982083 10.1093/cid/ciab416 PMC8528389

[52] Brittain K, Zerbe A, Phillips TK, et al Impact of adverse childhood experiences on women's psychosocial and HIV-related outcomes and early child development in their offspring. Glob Public Health 2022; 17:2779–91. 34613893 10.1080/17441692.2021.1986735 PMC8983791

[53] Hargreaves JR, Pliakas T, Hoddinott G, et al HIV stigma and viral suppression among people living with HIV in the context of universal test and treat: analysis of data from the HPTN 071 (PopART) trial in Zambia and South Africa. J Acquir Immune Defic Syndr 2020; 85:561–70. 32991336 10.1097/QAI.0000000000002504 PMC7654947

[54] Kemp CG, Lipira L, Huh D, et al HIV stigma and viral load among African-American women receiving treatment for HIV. AIDS 2019; 33:1511–9. 31259767 10.1097/QAD.0000000000002212 PMC6621603

[55] Lipira L, Williams EC, Huh D, et al HIV-Related Stigma and viral suppression among African-American women: exploring the mediating roles of depression and ART nonadherence. AIDS Behav 2019; 23:2025–36. 30343422 10.1007/s10461-018-2301-4 PMC6815932

[56] Zhu J, Exner-Cortens D, Dobson K, Wells L, Noel M, Madigan S. Adverse childhood experiences and intimate partner violence: a meta-analysis. Dev Psychopathol 2024; 36:929–43. 37009672 10.1017/S0954579423000196

[57] Mair C, Cunradi CB, Todd M. Adverse childhood experiences and intimate partner violence: testing psychosocial mediational pathways among couples. Ann Epidemiol 2012; 22:832–9. 23084843 10.1016/j.annepidem.2012.09.008 PMC3508260

[58] Masiano SP, Yu X, Tembo T, et al The relationship between adverse childhood experiences and common mental disorders among pregnant women living with HIV in Malawi. J Affect Disord 2022; 312:159–68. 35752220 10.1016/j.jad.2022.06.028 PMC9892657

[59] Onono M, Odwar T, Abuogi L, et al Effects of depression, stigma and intimate partner violence on postpartum Women's adherence and engagement in HIV care in Kenya. AIDS Behav 2020; 24:1807–15. 31813076 10.1007/s10461-019-02750-y PMC7228848

[60] Manongi R, Rogathi J, Sigalla G, et al The association between intimate partner violence and signs of depression during pregnancy in Kilimanjaro Region, Northern Tanzania. J Interpers Violence 2020; 35:5797–811. 29294866 10.1177/0886260517724256

[61] Matseke G, Rodriguez VJ, Peltzer K, Jones D. Intimate partner violence among HIV positive pregnant women in South Africa. J Psychol Afr 2016; 26:259–66. 27574487 PMC5001158

[62] Nyamukoho E, Mangezi W, Marimbe B, Verhey R, Chibanda D. Depression among HIV positive pregnant women in Zimbabwe: a primary health care based cross-sectional study. BMC Pregnancy Childbirth 2019; 19:53. 30704428 10.1186/s12884-019-2193-y PMC6357405

[63] Peltzer K, Rodriguez VJ, Lee TK, Jones D. Prevalence of prenatal and postpartum depression and associated factors among HIV-infected women in public primary care in rural South Africa: a longitudinal study. AIDS Care 2018; 30:1372–9. 29575943 10.1080/09540121.2018.1455960 PMC6150830

[64] Rodriguez VJ, Shaffer A, Lee TK, Peltzer K, Weiss SM, Jones DL. Psychological and physical intimate partner violence and maternal depressive symptoms during the Pre- and post-partum period among women living with HIV in rural South Africa. J Fam Violence 2020; 35:73–83. 32636575 10.1007/s10896-018-0027-8 PMC7339971

[65] Kuchukhidze S, Walters MK, Panagiotoglou D, et al The contribution of intimate partner violence to vertical HIV transmission: a modelling analysis of 46 African countries. Lancet HIV 2024; 11:e542–51. 39059403 10.1016/S2352-3018(24)00148-6 PMC12465746

[66] Koenig LJ, Khurana N, Islam MH, Gopalappa C, Farnham PG. Closing the gaps in the continuum of depression care for persons with HIV: modeling the impact on viral suppression in the United States. AIDS 2023; 37:1147–56. 36927810 10.1097/QAD.0000000000003536 PMC10986188

[67] US Preventive Services Task Force . Interventions to prevent perinatal depression: US preventive services task force recommendation statement. JAMA 2019; 321:580–7. 30747971 10.1001/jama.2019.0007

[68] Barry MJ, Nicholson WK, Silverstein M, et al Screening for depression and suicide risk in adults: US preventive services task force recommendation statement. JAMA 2023; 329:2057–67. 37338872 10.1001/jama.2023.9297

[69] Curry SJ, Krist AH, Owens DK, et al Screening for intimate partner violence, elder abuse, and abuse of vulnerable adults: US preventive services task force final recommendation statement. JAMA 2018; 320:1678–87. 30357305 10.1001/jama.2018.14741

[70] Feltner C, Wallace I, Berkman N, et al Screening for intimate partner violence, elder abuse, and abuse of vulnerable adults: evidence report and systematic review for the US Preventive Services Task Force. JAMA 2018; 320:1688–701. 30357304 10.1001/jama.2018.13212

[71] The American College of Obstetricians and Gynecologists . ACOG Committee Opinion No. 518: intimate partner violence. Obstet Gynecol 2012; 119:412–7. 22270317 10.1097/AOG.0b013e318249ff74

[72] The American College of Obstetricians and Gynecologists . ACOG Committee Opinion No. 757: screening for perinatal depression. Obstet Gynecol 2018; 132:e208–12. 30629567 10.1097/AOG.0000000000002927

[73] US Preventive Services Task Force . Intimate partner violence, elder abuse, and abuse of vulnerable adults: screening. Draft recommendation statement. **2024**. Updated October 29, 2024. Accessed November 1, 2024. Available at: https://www.uspreventiveservicestaskforce.org/uspstf/.

[74] Centers for Disease Control and Prevention; HIV Medicine Association of the Infectious Diseases Society of America; Pediatric Infectious Diseases Society; HHS Panel on Treatment of HIV During Pregnancy and Prevention of Perinatal Transmission—A Working Group of the Office of AIDS Research Advisory Council (OARAC). Recommendations for the use of antiretroviral drugs during pregnancy and interventions to reduce perinatal Hiv transmission in the United States Department Of Health And Human Services. Rockville, MD: US Department of Health and Human Services, 2024.

[75] Mendez NA, Mayo D, Safren SA. Interventions addressing depression and HIV-related outcomes in people with HIV. Curr HIV/AIDS Rep 2021; 18:377–90. 34014446 10.1007/s11904-021-00559-w PMC8136266

[76] Sherr L, Clucas C, Harding R, Sibley E, Catalan J. HIV and depression–a systematic review of interventions. Psychol Health Med 2011; 16:493–527. 21809936 10.1080/13548506.2011.579990

[77] Alexander KA, Mpundu G, Duroseau B, et al Intervention approaches to address intimate partner violence and HIV: a scoping review of recent research. Curr HIV/AIDS Rep 2023; 20:296–311. 37768511 10.1007/s11904-023-00668-8

[78] Andersson GZ, Reinius M, Eriksson LE, et al Stigma reduction interventions in people living with HIV to improve health-related quality of life. Lancet HIV 2020; 7:e129–40. 31776098 10.1016/S2352-3018(19)30343-1 PMC7343253

[79] Hackett S, Badell ML, Meade CM, et al Improved perinatal and postpartum human immunodeficiency virus outcomes after use of a perinatal care coordination team. Open Forum Infect Dis 2019; 6:ofz183. 31198816 10.1093/ofid/ofz183 PMC6545466

[80] Willie TC, Stockman JK, Perler R, Kershaw TS. Associations between intimate partner violence, violence-related policies, and HIV diagnosis rate among women in the United States. Ann Epidemiol 2018; 28:881–5. 30055935 10.1016/j.annepidem.2018.07.008 PMC6286221

[81] Sullivan PS, Johnson S, Pembleton A, et al Epidemiology of HIV in the USA: epidemic burden, inequities, contexts, and responses. Lancet 2021; 397:1095–106. 33617774 10.1016/S0140-6736(21)00395-0 PMC12707558

[82] Fleszar LG, Bryant AS, Johnson CO, et al Trends in state-level maternal mortality by racial and ethnic group in the United States. JAMA 2023; 330:52–61. 37395772 10.1001/jama.2023.9043 PMC10318476

[83] Tran N, Callaway L, Shen S, et al Screening for adverse childhood experiences in antenatal care settings: a scoping review. Aust N Z J Obstet Gynaecol 2022; 62:626–34. 35909247 10.1111/ajo.13585 PMC9796324

[84] Castillo-Mancilla JR, Morrow M, Hunt PW, et al Beyond undetectable: modeling the clinical benefit of improved antiretroviral adherence in persons with Human Immunodeficiency Virus with virologic suppression. Open Forum Infect Dis 2023; 10:ofad230. 37213424 10.1093/ofid/ofad230 PMC10199113

