# Supplementary material for: Reduced Adherence to Antiretroviral Therapy in Pregnant Women With HIV With Intimate Partner Violence in the United States
Source: Open Forum Infect Dis. 2026 Jan 23;13(1):ofaf787. doi: 10.1093/ofid/ofaf787 (PMC12828428; doi:10.1093/ofid/ofaf787)
Supplement: ofaf787_Supplementary_Data [file ofaf787_supplementary_data.docx]

# Supplement

*Survey Questions for Psychosocial Variables*

**Intimate Partner Violence (IPV) questions from the World Health Organization (WHO) Violence Against Women instrument**

Participants responded to the following questions with response options: “Never”, “Once”, “2-3 times”, “4-10 times”, “More than 10 times”. The variable was dichotomized into any exposure to IPV (once or more than once) versus no exposure (never). Each question was asked for both during pregnancy and before pregnancy timeframes.

| Variable | Questions |
| --- | --- |
| Psychological IPV | • Has your partner insulted you or made you feel bad about yourself? • Has your partner belittled or humiliated you in front of other people? • Has your partner done things to scare or intimidate you on purpose (e.g. by the way he looked at you, by yelling and smashing things)? • Has your partner threatened to hurt you or someone you care about? |
| Physical IPV | • Has your partner slapped or thrown something at you that could hurt you? • Has your partner pushed or shoved you? • Has your partner hit you with his fist or something else that could hurt you? • Has your partner kicked you, dragged you or beaten you up? • Has your partner choked or burnt you on purpose? |
| Sexual IPV | • Has your partner physically forced you to have sex when you did not want to? • Has your partner used threats to make you have sex? • Has your partner forced you to do something sexual that you found degrading or humiliating? |

**Adverse Childhood Experiences (ACE) questions from the Behavioral Risk Factor Surveillance System survey (CDC)**

ACE scores were calculated by totaling the survey items, with 1 point assigned for each ACE (range 0-11).

Participants responded "yes" or "no" to the following questions about experiences during their first 18 years of life, with one point assigned for each "yes" response.

1. While you were growing up, during your first 18 years of life did you live with anyone who was depressed, mentally ill, or suicidal?
2. While you were growing up, during your first 18 years of life did you live with anyone who was a problem drinker or alcoholic?
3. While you were growing up, during your first 18 years of life did you live with anyone who used illegal street drugs or who abused prescription medications?
4. While you were growing up, during your first 18 years of life did you live with anyone who served time or was sentenced to serve time in a prison, jail, or other correctional facility?
5. While you were growing up, during your first 18 years of life were your parents separated or divorced?

Participants responded to the following questions about experiences during their first 18 years of life with response options: "Never", "Once", "More than once", "Don't know/Not sure". One point was assigned for the responses "Once" or "More than once"."

1. While you were growing up, during your first 18 years of life, how often did your parents or adults in your home ever slap, hit, kick, punch or beat each other up?
2. While you were growing up, during your first 18 years of life, how often did a parent or adult in your home ever hit, beat, kick, or physically hurt you in any way? Do not include spanking.
3. While you were growing up, during your first 18 years of life, how often did a parent or adult in your home ever swear at you, insult you, or put you down?
4. While you were growing up, during your first 18 years of life, how often did anyone at least 5 years older than you or an adult, ever touch you sexually?
5. While you were growing up, during your first 18 years of life, how often did anyone at least 5 years older than you or an adult, try to make you touch them sexually?
6. While you were growing up, during your first 18 years of life, did anyone at least 5 years older than you or an adult, force you to have sex?

**Depression questions from the Edinburgh Postnatal Depression Scale (EPDS)**

The EPDS comes from 10 questions scored from 0 to 3 depending on the severity of depression symptoms. Participants responded to the following questions about experiences in the past 7 days. Response options varied by question.

1. In the past 7 days, I have been able to laugh and see the funny side of things:

- As much as I always could (0)
- Not quite so much now (1)
- Definitely not so much now (2)
- Not at all (3)

1. In the past 7 days, I have looked forward with enjoyment to things:

- As much as I ever did (0)
- Rather less than I used to (1)
- Definitely less than I used to (2)
- Hardly at all (3)

1. In the past 7 days, I have blamed myself unnecessarily when things went wrong:

- Yes, most of the time (3)
- Yes, some of the time (2)
- Not very often (1)
- No, never (0)

1. In the past 7 days, I have been anxious or worried for no good reason:

- No, not at all (0)
- Hardly ever (1)
- Yes, sometimes (2)
- Yes, very often (3)

1. In the past 7 days, I have felt scared or panicky for no good reason:

- Yes, quite a lot (3)
- Yes, sometimes (2)
- No, not much (1)
- No, not at all (0)

1. In the past 7 days, things have been getting to me:

- Yes, most of the time I haven't been able to cope at all (3)
- Yes, sometimes I haven't been coping as well as usual (2)
- No, most of the time I have coped quite well (1)
- No, I have been coping as well as ever (0)

1. In the past 7 days, I have been so unhappy that I have had difficulty sleeping:

- Yes, most of the time (3)
- Yes, sometimes (2)
- Not very often (1)
- No, not at all (0)

1. In the past 7 days, I have felt sad or miserable: Y

- Yes, most of the time (3)
- Yes, quite often (2)
- Not very often (1)
- No, not at all (0)

1. In the past 7 days, I have been so unhappy that I have been crying:

- Yes, most of the time (3)
- Yes, quite often (2)
- Only occasionally (1)
- No, never (0)

1. In the past 7 days, the thought of harming myself has occurred to me:

- Yes, quite often (3)
- Sometimes (2)
- Hardly ever (1)
- Never (0)
